# Relationship between high platelet reactivity on clopidogrel and long-term clinical outcomes after drug-eluting stents implantation (PAINT-DES): a prospective, propensity score-matched cohort study

**DOI:** 10.1186/s12872-018-0841-1

**Published:** 2018-05-24

**Authors:** Xiao-Fei Gao, Shu Lu, Zhen Ge, Guang-Feng Zuo, Zhi-Mei Wang, Feng Wang, Xiang-Quan Kong, Da-Yang Chai, Shao-Liang Chen, Jun-Jie Zhang

**Affiliations:** 1 Department of Cardiology, Nanjing First Hospital, Nanjing Medical University, No. 68 Changle Road, Nanjing, 210006 China; 2 Department of Cardiology, The First People’s Hospital of Taicang, Suzhou, China; 3 Department of Cardiology, Nanjing Heart Center, Nanjing, China

**Keywords:** Platelet function testing, Clopidogrel, High platelet reactivity, Drug eluting stent

## Abstract

**Background:**

The relationship between platelet reactivity and long-term clinical outcomes remains controversial. The present prospective study was designed to explore the association between high platelet reactivity (HPR) on clopidogrel and long-term clinical outcomes following implantation of drug eluting stents (DES).

**Methods:**

A total of 1769 consecutive patients assessed by Aggrestar (PL-11) were enrolled at our center from February 2011 to December 2017. The primary end point was major adverse cardiovascular and cerebrovascular events (MACCE), defined as definite or probable stent thrombosis, spontaneous myocardial infarction, all cause death, clinically driven target vessel revascularization (TVR), or ischemic stroke. Bleeding served as the safety endpoint. Propensity score matching (PSM) analysis was performed to adjust for baseline differences in the overall cohort.

**Results:**

Finally, 409 patients (23.1%) were identified with HPR on clopidogrel. At a median follow-up of 4.1 years (interquartile range, 1.8 years), the occurrence of MACCE was significantly higher in HPR on clopidogrel group than normal platelet reactivity (NPR) on clopidogrel group (15.6% vs. 5.4%, *p* < 0.001). After PSM, 395 paired patients were matched, and the difference in MACCE between HPR (15.7%) versus NPR (9.4%) on clopidogrel groups remained significant (*P* < 0.001), mainly driven by increased all cause death (5.3% vs. 1.8%, *p* < 0.001), and clinically driven TVR (8.1% vs. 6.3%, *p* = 0.019) in the HPR group. The risk of bleeding between two groups was similar.

**Conclusions:**

This prospective study confirms the relationship between HPR on clopidogrel and long-term adverse cardiovascular events after coronary stenting.

**Electronic supplementary material:**

The online version of this article (10.1186/s12872-018-0841-1) contains supplementary material, which is available to authorized users.

## Background

Dual antiplatelet therapy with aspirin and clopidogrel, an adenosine diphosphate (ADP)-receptor inhibitor, is widely prescribed for patients with percutaneous coronary intervention (PCI) to prevent stent thrombosis, which has a high risk of myocardial infarction (MI) and cardiac death [1, 2]. However, patients treated with protocolized aspirin and clopidogrel may still develop adverse cardiovascular events, and high platelet reactivity (HPR) on clopidogrel (clopidogrel no-response or clopidogrel resistance) has been thought to play a critical role in their occurrence [3].

In platelet function testing studies [4, 5], the rate of HPR on clopidogrel was 4–46% in patients on conventional dose of clopidogrel. Randomized clinical trials (RCTs) [6–9] failed to show benefit of antiplatelet regimen adjustment according to platelet function testing for coronary stenting, which might partly be due to exclusion of high-risk patients, and randomization performed a few hours after PCI. The current consensus [10] from large observational studies [3, 11–14] is that HPR on clopidogrel is associated with increased short-term risk of cardiac events following drug-eluting stents (DES) implantation. However, there is scarce evidence on the relationship between HPR on clopidogrel and long-term cardiovascular events in PCI patients. Therefore, the present prospective, propensity score-matched (PSM) cohort study explored the long-term impact of HPR on clopidogrel in patients with DES implantation.

## Methods

### Study design and population

The PAINT-DES is a prospective, single-center cohort study designed to assess the association between HPR on clopidogrel and long-term cardiovascular events in patients with DES implantation. From February 2011 to December 2017, a total of 6200 consecutive real-word PCI patients at our center with loading dose of aspirin and clopidogrel were candidates for this study. Finally, 1769 patients with platelet function testing were enrolled in the present study if they met the following inclusion criteria: age > 18 years, and at least one successful DES implantation. The major exclusion criteria were: patients on ticagrelor or oral anticoagulation (warfarin, dabigatran, rivaroxaban, etc.); platelet count < 10 ×  10^9^ /L; acute myocardial infarction < 24 h; suspected intolerance to study drugs; major complication during PCI; and an estimated life expectancy < 12 months (Additional file 1: Figure S1). Cases missing > 10% of baseline variables also were excluded. The study protocol and informed consent were reviewed and approved by the Institutional Review Board of Nanjing First Hospital. Written informed consent for participation was obtained from all enrolled patients.

### Procedure and medication

All coronary interventions were performed according to current standards. Use of coronary imaging tools, type of DES, low molecular weight heparin, glycoprotein IIb/IIIa inhibitors, and intra-aortic balloon pump counterpulsation were at operator’s discretion. Patients not on aspirin 100 mg daily for more than 7 days were loaded with 300 mg at least 6 h prior to index procedure. A loading dose of clopidogrel of 300 mg or 600 mg was prescribed at least 6 h before the procedure, followed by a daily maintenance dose of 75 mg. Total creatine kinase (CK), CK-myocardial-band isoenzyme (MB), and troponin T/I were dynamically measured before the procedure and until 72 h post-procedure. After the procedure, all patients were prescribed aspirin 100 mg daily indefinitely and clopidogrel 75 mg daily for at least 12 months.

### Platelet function testing

Platelet function testing with Aggrestar (PL-11) analyzer (SINNOWA Co., Nanjing, China) was performed before heparin or antithrombotic agent administration during the index procedure in the cardiac catheterization lab. Blood samples were stored at room temperature and tested within 2 h after collection. Information on the Aggrestar analyzer has been provided in detail before [12, 15]. Briefly, platelet function was tested using sequentially platelet counting method, with automatically and sequentially counting platelet numbers in the citrated whole blood sample before and after adding agonists at fixed time intervals. Aggrestar analyzer provides more stable and accurate platelet maximum aggregation ratio (MAR%) than standardized test method. HPR on clopidogrel was defined as MAR% ≥ 55% according to a previous study [12]. It was not recommended to adjust antiplatelet strategy based on the results of platelet function testing.

### Endpoints and definitions

The primary endpoint was rate of major adverse cardiovascular and cerebrovascular events (MACCE), defined as definite or probable stent thrombosis, spontaneous MI, all cause death, clinically driven target vessel revascularization (TVR), or ischemic stroke. The secondary endpoints included stent thrombosis, cardiac death, clinically driven target lesion revascularization (TLR), and individual components of the primary endpoint. Spontaneous MI was diagnosed based on Third Universal Definition of Myocardial Infarction [16]. Cardiac death was defined as any death without a clear non-cardiac cause. Clinically driven TLR or TVR was defined as angina or ischemia referable to the target lesion or target vessel requiring repeated PCI or bypass surgery. Ischemic stroke was defined as neurological deficit of cerebrovascular cause according to the related symptoms and physical examination and brain imaging. Bleeding served as the safety endpoint, defined in accordance to the Bleeding Academic Research Consortium (BARC) classification [17]. Stent thrombosis was defined based on the Academic Research Consortium (ARC) classification [18]. Lesion specificities were defined based on American Heart Association/American College of Cardiology criteria [19]. Complex coronary lesions in this study were defined as one of the following: multi-vessel lesions, moderate to severe calcification, chronic total occlusion, coronary bifurcations treated with two-stent techniques, and unprotected left main lesions.

### Follow up

Clinical follow-up was conducted with visits (preferred) or telephone contact at 1, 6, 12 months, and yearly thereafter. Angiographic follow-up was scheduled for all patients at 13 months after PCI, unless clinically indicated earlier. An independent committee that was blinded to the study assessed all adverse clinical events.

### Statistical analysis

Continuous baseline variables are expressed as means ± standard deviation (SD) or median (interquartile range) and were compared using Students’ *t*-test for normal data and Wilcoxon rank sum scores for non-normally distributed data. Categorical variables are expressed as counts and percentages and were compared by Chi-square test or Fisher’s exact test. Time-to-first event curves between two groups were generated by Kaplan-Meier analysis and compared by a log-rank test. For significant differences (*p* < 0.10) in baseline clinical characteristics (age, male, body mass index [BMI], hypertension, hyperlipidemia, current smoker, prior stroke, prior MI, and prior PCI), and procedural characteristics (chronic total occlusion, and complete revascularization) between two groups, propensity score matching at a ratio of 1:1 was performed to minimize any selection bias using logistic regression according to the nearest rule, with matching tolerance of 0.01. Potential interactions between the subgroups and platelet reactivity were examined for the primary outcome. Multivariable Cox proportional hazards forward stepwise regression was performed to determine independent predictors of the primary endpoint with purposeful selection of covariates. The variables showing possible statistical significance (*P* < 0.10) in univariable model and those judged to be of clinical importance from previous studies (age, male, body mass index, hypertension, diabetes, current smoker, complex lesions, stent number, intravascular ultrasound used, and complete revascularization) were entered into the Cox multivariable model. All statistical tests were two-sided, and a *p*-value of < 0.05 was considered statistically significant. All statistical analyses were performed with SPSS version 24.0 (SPSS Institute Inc., Chicago, Illinois, USA).

## Results

### Baseline clinical characteristics

Of 1769 patients with DES implantation, 409 (23.1%) were in the HPR on clopidogrel group, and the remainder was in the normal platelet reactivity (NPR) on clopidogrel group. The baseline clinical characteristics are summarized in Table 1. Notably, patients in the HPR on clopidogrel group were older (66.9 ± 10.2), and with higher BMI (25.1 ± 3.6 kg/mm^2^), and more frequent hypertension (76.0%), hyperlipidemia (49.9%), and prior stroke (3.7%) when compared with the NPR on clopidogrel group (64.6 ± 10.2, *p* < 0.001; 24.6 ± 2.9 kg/mm2, *p* = 0.003; 69.8%, *p* = 0.014; 38.3%, *p* < 0.001; 1.3%, *p* = 0.005, respectively). They also were less often men (70.7% versus 75.6%, *p* = 0.045). All the other baseline variables were similarly distributed.

**Table 1:** Baseline clinical characteristics

	Before propensity score matching			After propensity score matching		
	NPR on clopidogrel (*n* = 1360)	HPR on clopidogrel (*n* = 409)	*P* Value	NPR on clopidogrel (*n* = 395)	HPR on clopidogrel (*n* = 395)	*P* Value
Age, year	64.6 ± 10.2	66.9 ± 10.2	< 0.001	66.5 ± 9.8	66.7 ± 10.2	0.765
Male, n (%)	1028 (75.6)	289 (70.7)	0.045	264 (66.8)	278 (70.4)	0.283
BMI, kg/mm^2^	24.6 ± 2.9	25.1 ± 3.6	0.003	25.3 ± 3.1	25.0 ± 3.1	0.169
Hypertension, n (%)	949 (69.8)	311 (76.0)	0.014	298 (75.4)	305 (77.2)	0.558
Hyperlipidemia, n (%)	521 (38.3)	204 (49.9)	< 0.001	198 (50.1)	200 (50.6)	0.887
Diabetes, n (%)	372 (27.4)	127 (31.1)	0.145	118 (29.9)	125 (31.6)	0.589
Current smoker, n (%)	511 (37.6)	132 (32.3)	0.051	137 (34.7)	131 (33.2)	0.652
ACS, n (%)	1226 (90.1)	361 (88.3)	0.272	359 (90.9)	349 (88.4)	0.243
eGFR ≥60 ml/min/1.73m^2^, n(%)	1205 (88.9)	359 (88.0)	0.625	339 (86.5)	347 (88.1)	0.503
Prior stroke, n (%)	18 (1.3)	15 (3.7)	0.005	5 (1.3)	12 (3.0)	0.086
Prior MI, n (%)	124 (9.1)	49 (12.0)	0.088	42 (10.6)	48 (12.2)	0.502
Prior PCI, n (%)	267 (19.6)	96 (23.5)	0.092	76 (19.2)	96 (24.3)	0.085
Prior CABG, n (%)	12 (0.9)	6 (1.5)	0.397	3 (0.8)	6 (1.5)	0.505
LVEF, %	59.7 ± 9.0	58.4 ± 8.2	0.488	58.5 ± 9.4	58.4 ± 8.2	0.952
Symptomatic HF, n (%)	222 (16.5)	79 (19.6)	0.155	97 (24.6)	79 (20.0)	0.124
Treatment at discharge						
Aspirin maintenance dose, n (%)	1360 (100)	408 (99.8)	0.231	395 (100)	394 (99.7)	0.317
Clopidogrel maintenance dose of 75 mg, n (%)	1331 (97.9)	395 (96.6)	0.137	381 (96.5)	381 (96.5)	NS
Clopidogrel maintenance dose of 150 mg, n (%)	29 (2.1)	14 (3.4)	0.137	14 (3.5)	14 (3.5)	NS
Treatment at last follow-up visit						
Aspirin maintenance dose, n (%)	1292 (95)	386 (94.4)	0.617	376 (95.2)	372 (94.2)	0.526
Clopidogrel maintenance dose, n (%)	494 (36.3)	140 (34.2)	0.439	132 (33.4)	127 (32.2)	0.705
Ticagrelor maintenance dose, n (%)	11 (0.8)	5 (1.2)	0.388	6 (1.5)	4 (1.0)	0.524
Laboratory						
Red blood cell, × 10^12^/L	4.4 ± 0.6	4.6 ± 6.5	0.314	4.3 ± 0.6	4.6 ± 6.5	0.596
Hemoglobin, g/L	133.7 ± 17.2	132.1 ± 55.3	0.436	132.0 ± 17.9	132.2 ± 55.8	0.973
Platelet count, × 10^9^/L	190.6 ± 66.9	190.3 ± 51.5	0.937	186.1 ± 55.5	189.9 ± 51.1	0.431
hsCRP, ug/ml	32.1 ± 8.9	31.9 ± 8.8	0.858	31.8 ± 8.6	31.9 ± 8.9	0.883

*NPR* normal platelet reactivity, *HPR* high platelet reactivity, *BMI* body mass index, *ACS* acute coronary syndrome, *eGFR* estimated glomerular filtration rate, *MI* myocardial infarction, *PCI* percutaneous coronary intervention, *CABG* coronary artery bypass grafting, *LVEF* left ventricular ejection fraction, *HF* heart failure, *hsCRP* high sensitivity C reactive protein

### Lesion and procedural characteristics

Table 2 shows that patients in the HPR on clopidogrel group had more frequent chronic total occlusion (15.4%) compared with the NPR on clopidogrel group (10.0%, *p* = 0.003). Moreover, there was a numerically lower rate of complete revascularization for all diseased coronary segments (defined as ≥2.5 mm in diameter by visual estimation) in the HPR on clopidogrel group than that in the NPR on clopidogrel group (59.6% vs. 64.8%, *p* = 0.054). All the other angiographic and procedural characteristics were comparable between two groups.

**Table 2:** Angiographic and procedural characteristics

	Before propensity score matching^a^			After propensity score matching		
	NPR on clopidogrel (*n* = 1360)	HPR on clopidogrel (*n* = 409)	*P* Value	NPR on clopidogrel (*n* = 395)	HPR on clopidogrel (*n* = 395)	*P* Value
Radial access, n (%)	1249 (92.9)	372 (92.1)	0.596	355 (89.9)	367 (92.9)	0.128
Multi-vessel disease, n (%)	605 (46.4)	200 (49.5)	0.280	188 (50.4)	196 (51.0)	0.860
Bifurcation lesion, n (%)	410 (30.5)	135 (33.4)	0.268	129 (32.7)	133 (33.7)	0.762
Chronic total occlusion, n (%)	133 (10.0)	63 (15.4)	0.003	64 (16.2)	62 (15.7)	0.846
Unprotected Left main lesions, n (%)	146 (10.9)	46 (11.4)	0.765	51 (12.9)	45 (11.4)	0.514
Second-generation DES used, n (%)	1360 (100)	409 (100)	NS	395 (100)	395 (100)	NS
IABP used, n (%)	23 (1.8)	6 (1.6)	0.753	13 (3.5)	6 (1.6)	0.108
IVUS used, n (%)	291 (22.9)	100 (26.1)	0.189	79 (21.2)	97 (26.2)	0.106
Stent number	1.9 ± 1.1	1.9 ± 1.1	0.328	1.9 ± 1.1	1.9 ± 1.1	0.946
Mean stent diameter, mm	3.0 ± 0.7	2.9 ± 0.7	0.770	3.0 ± 0.6	2.9 ± 0.7	0.166
Stent length, mm	49.1 ± 31.2	51.0 ± 30.4	0.299	49.6 ± 31.1	50.6 ± 30.2	0.650
Complete revascularization, n (%)	852 (64.8)	240 (59.6)	0.054	252 (63.8)	237 (60.0)	0.272
Final TIMI grade 3, n (%)	1301 (98.3)	393 (97.5)	0.339	387 (98.5)	384 (97.5)	0.315

^a^ There is some data loss in dozens of patients

*NPR* normal platelet reactivity, *HPR* high platelet reactivity, *DES* drug-eluting stent, *IABP* intra-aortic balloon pump, *IVUS* intravascular ultrasound, *TIMI* thrombolysis in myocardial infarction

### Unadjusted clinical outcomes

Clinical follow-up was available in 94.7% of all patients, and angiographic follow-up was conducted in 70.1% of patients. Unadjusted clinical outcomes are listed in Table 3. After a median follow-up of 4.1 years (interquartile range, 1.8 years), the incidence of definite and probable stent thrombosis, all cause death, clinically driven TVR, and ischemic stroke in the HPR on clopidogrel group was 1.5, 5.1, 7.8, and 2.2%, respectively, which was significantly higher than 0.3% (*p* = 0.003), 1.3% (*p* < 0.001), 3.2% (*p* < 0.001), and 0.5% (p < 0.001), respectively, in the NPR on clopidogrel group, resulting in more frequent MACCE in the HPR on clopidogrel group (15.6% vs. 5.4%, *p* < 0.001). The occurrence of bleeding (BARC classification ≥2) was 2.9% in the HPR on clopidogrel group, which was comparable with 2.6% in the NPR on clopidogrel group (*p* = 0.754).

**Table 3:** Clinical outcomes

	Before propensity score matching			After propensity score matching		
	NPR on clopidogrel (*n* = 1360)	HPR on clopidogrel (*n* = 409)	*P* Value	NPR on clopidogrel (*n* = 395)	HPR on clopidogrel (*n* = 395)	*P* Value
Stent thrombosis	7 (0.5)	13 (3.2)	< 0.001	3 (0.8)	13 (3.3)	0.003
Definite/ probable Stent thrombosis	4 (0.3)	6 (1.5)	0.003	2 (0.5)	6 (1.5)	0.110
All cause death	18 (1.3)	21 (5.1)	< 0.001	7 (1.8)	21 (5.3)	< 0.001
Cardiac death	6 (0.4)	10 (2.4)	0.001	2 (0.5)	10 (2.5)	0.004
MI	12 (0.9)	7 (1.7)	0.085	6 (1.5)	7 (1.8)	0.526
STEMI	4 (0.3)	5 (1.2)	0.015	2 (0.5)	5 (1.3)	0.215
NSTEMI	10 (0.7)	3 (0.7)	NS	5 (1.3)	3 (0.8)	0.734
Clinically driven TLR	40 (2.9)	26 (6.4)	< 0.001	24 (6.1)	26 (6.6)	0.111
Clinically driven TVR	43 (3.2)	32 (7.8)	< 0.001	25 (6.3)	32 (8.1)	0.019
Ischemic stroke	7 (0.5)	9 (2.2)	< 0.001	4 (1.0)	7 (1.8)	0.093
Bleeding (BARC≥2)	36 (2.6)	12 (2.9)	0.754	11 (2.8)	11 (2.8)	NS
MACCE	74 (5.4)	64 (15.6)	< 0.001	37 (9.4)	62 (15.7)	< 0.001

Data are number of events (Kaplan-Meier estimated event rate), compared by the log-rank test

*NPR* normal platelet reactivity, *HPR* high platelet reactivity, *MI* myocardial infarction, *TLR* target lesion revascularization, *TVR* target vessel revascularization, *MACCE* major adverse cardiovascular and cerebrovascular events

### Propensity score matched analysis

**Fig. 1:**
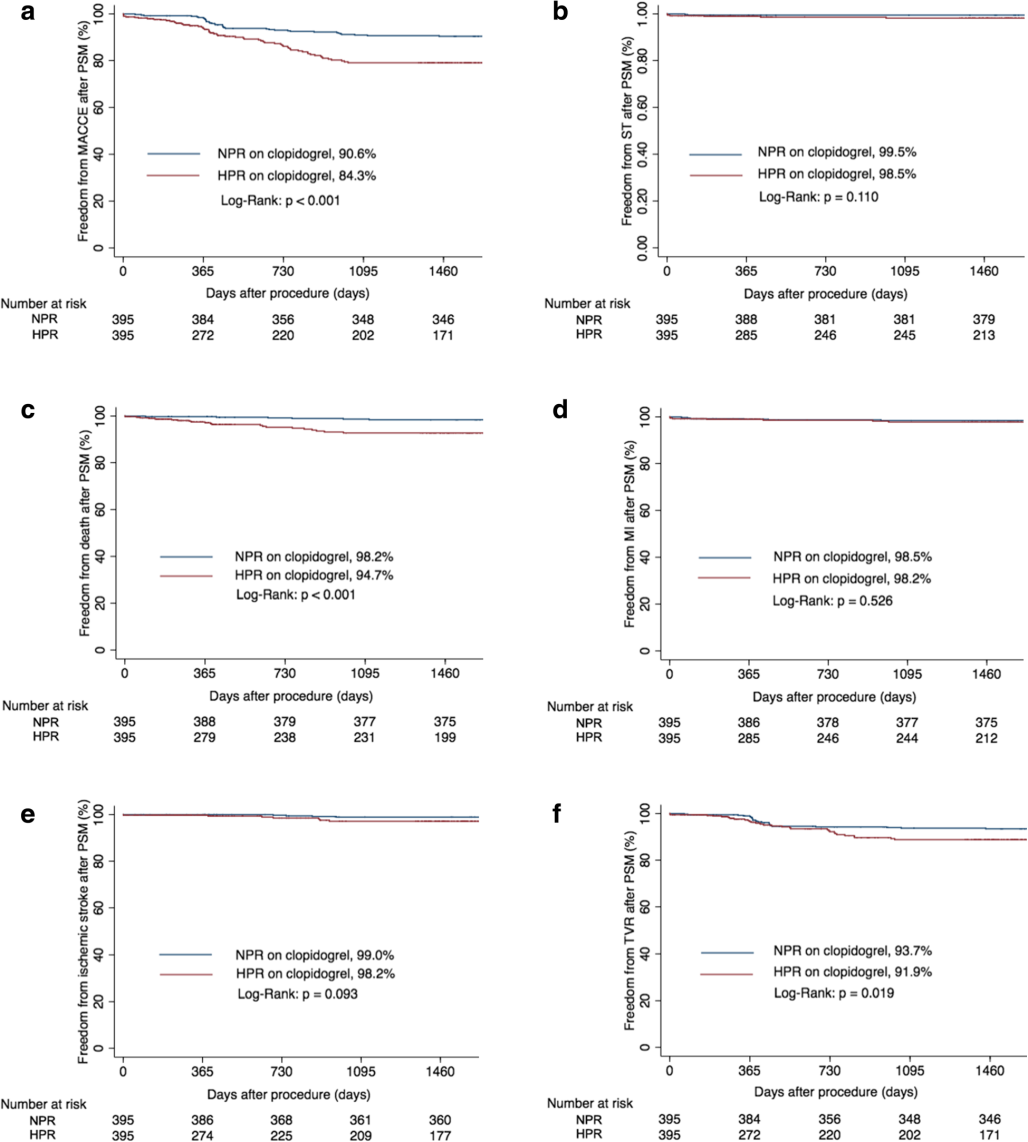
Freedom from events in the propensity score-matched population. Freedom from major adverse cardiovascular and cerebrovascular events (MACCE) (**a**), definite/ probable stent thrombosis (ST) (**b**), all cause death (**c**), myocardial infarction (MI) (**d**), ischemic stroke (**e**), and clinically driven target vessel revascularization (TVR) (**f**) between high platelet reactivity (HPR) on clopidogrel and normal platelet reactivity (NPR) on clopidogrel in the propensity score-matched (PSM) population

**Fig. 2:**
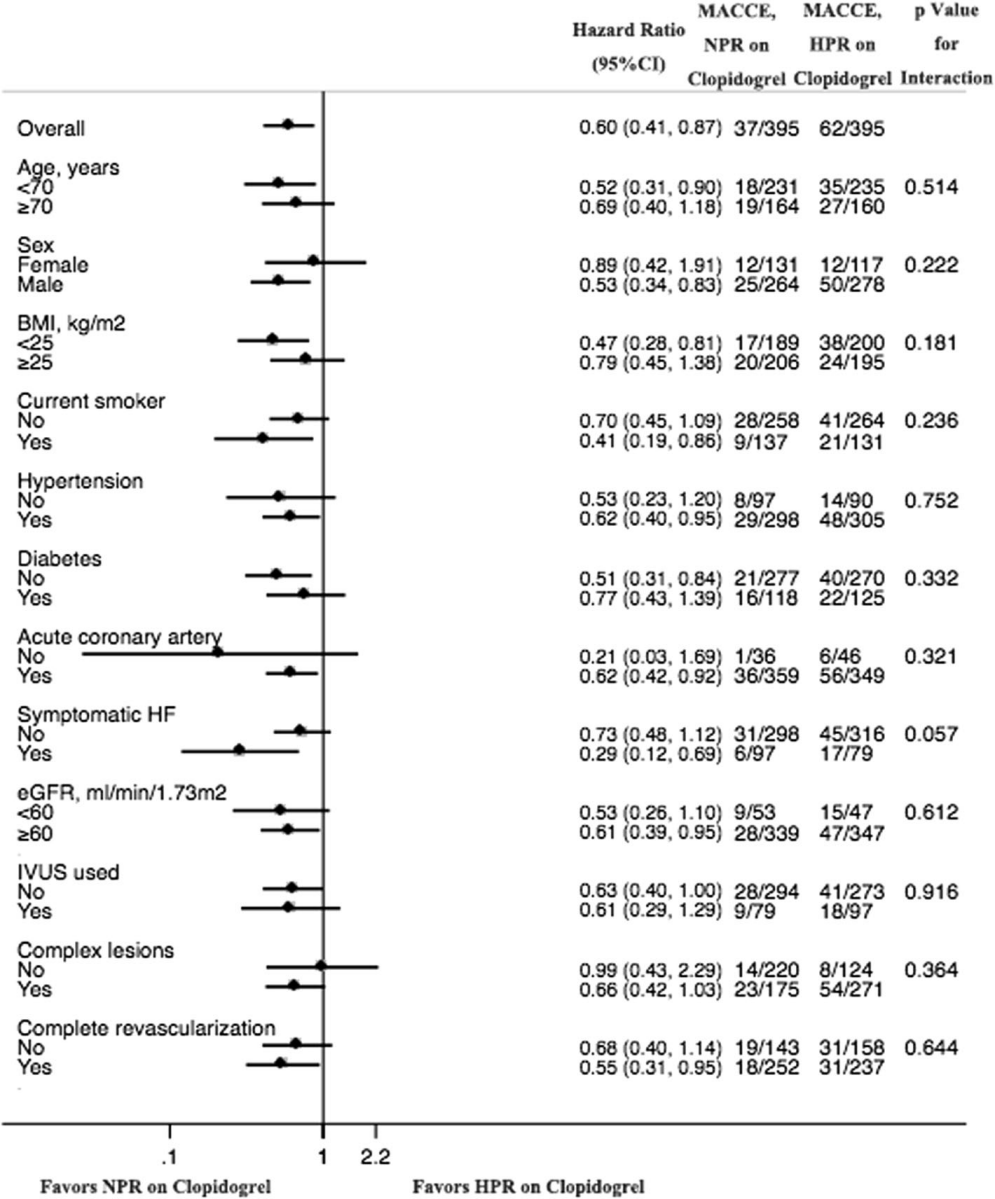
Subgroup analysis. The increment in major adverse cardiovascular and cerebrovascular events (MACCE) with high platelet reactivity (HPR) on clopidogrel compared with normal platelet reactivity (NPR) on clopidogrel was consistent across pre-specified subgroups. BMI: body mass index; HF: heart failure; eGFR: estimated glomerular filtration rate; IVUS: intravascular ultrasound

After PSM, 395 pairs of patients were matched. The baseline characteristics became comparable between two groups (Tables 1 and 2). There was still a significant difference in MACCE between the NPR on clopidogrel group and the HPR on clopidogrel group (9.4% vs. 15.7%, *p* < 0.001; Table 3, Fig. 1), mainly driven by increased all cause death (1.8% vs. 5.3%, *p* < 0.001), and clinically driven TVR (6.3% vs. 8.1%, *p* = 0.019) in the latter group. The risk of bleeding (BARC classification≥2) between these two groups remained similar. There were no significant interactions between any of the subgroups and platelet reactivity for MACCE (Fig. 2). In Cox regression multivariable analysis, diabetes (hazard ratio [HR]: 1.566, 95% confidence interval [CI]: 1.028–2.385, *p* = 0.037), HPR on clopidogrel (HR: 2.146, 95% CI: 1.387–3.320, *p* = 0.001), and complex coronary lesions (HR: 2.510, 95% CI: 1.522–4.140, *p* < 0.001) were independent predictors of MACCE at a median follow-up of 4.1 years.

## Discussion

The present prospective, propensity score-matched cohort study for the first time evaluated the relationship between HPR on clopidogrel and long-term outcomes following DES implantation. The major findings were: 1) 23.1% of patients with DES implantation were identified with HPR on clopidogrel despite having received loading dose of clopidogrel; 2) HPR on clopidogrel was associated with dramatic increment in unadjusted and adjusted long-term MACCE, mainly driven by increased all cause death and clinically driven TVR; and 3) there was no significant association between platelet reactivity and unadjusted and adjusted risk of bleeding.

Antiplatelet agents, aspirin and clopidogrel, have a critical role in the treatment of coronary heart disease, ischemic stroke and peripheral artery disease. However, stented patients suffer from recurrent thrombotic events despite being on regular aspirin and clopidogrel, which might be due to HPR on clopidogrel. Several factors [20–22] might underlie HPR on clopidogrel including patient noncompliance, intestinal absorption (ABCB1 gene polymorphism), polymorphisms in CYP2C19, drug-drug interactions (e.g. omeprazole, β-Blockers [23]), and other clinical factors (age, BMI, diabetes, chronic renal insufficiency, heart failure). More potent ADP-receptor inhibitors, such as ticagrelor and prasugrel, were expected to overcome HPR on clopidogrel because they showed better clinical outcomes than clopidogrel in patients with acute coronary syndrome (ACS) [24, 25]. However, ticagrelor and prasugrel were associated with higher risk of major bleeding [24–26]. Furthermore, recent studies [27, 28] demonstrated that clopidogrel was superior to ticagrelor to prevent bleeding complications without increased risk of ischemic events in low-to-moderate risk of ACS, especially among those treated with newer-generation DES. Overall, clopidogrel, with low cost and low bleeding risk, is still widely used in clinical practice, and platelet reactivity is worth exploring further.

The optimal method for platelet function testing is still a key and controversial issue. Although various methods have been developed, their results correlate poorly with each other [29, 30]. Light transmittance aggregation (LTA) is considered the gold standard, but it is time and labor consuming. VerifyNow system has been widely used in clinical trials, but it is very expensive and nonflexible [31, 32]. As described in the methods section, Aggrestar analyzer provides more stable and accurate platelet numbers, with good correlation with VerifyNow [15], which renders it a reliable alternative for platelet function testing.

Individualized treatment based on platelet function testing was expected to inform choice of more suitable antiplatelet agents and dosage for stenting patients, balancing the risk of ischemia and bleeding. In line with our previous study [12], HPR on clopidogrel was associated with increased risk for stent thrombosis in a prospective multicenter study [14]. The present prospective study with propensity-matched population is the first to confirm the relationship between HPR on clopidogrel and long-term thrombotic events in patients with DES implantation. In contrast to these findings, ARCTIC study (Bedside Monitoring to Adjust Antiplatelet Therapy for Coronary Stenting), including 2440 patients scheduled for coronary stenting, showed no significant improvements in clinical outcomes with platelet function testing and treatment adjustment for coronary stenting [7]. The latter difference might be due to the higher proportion of patients at higher risk in our study, with higher frequency of diabetes, ACS, prior MI, prior PCI, symptomatic heart failure, and complex coronary lesions. However, TROPICAL-ACS trial (The Testing Responsiveness To Platelet Inhibition On Chronic Antiplatelet Treatment For ACS) demonstrated that platelet function testing guided de-escalation of antiplatelet treatment (from prasugrel to clopidogrel) was non-inferior to standard treatment with prasugrel at 1 year after PCI, providing important evidence for ACS patients with coronary stenting in whom early de-escalation is considered as an alternative strategy [33]. Notably, the recent CREATIVE trial [34] (Clopidogrel Response Evaluation and AnTi-platelet InterVEntion in High Thrombotic Risk PCI Patients) also showed that intensified antiplatelet strategies with adjunctive use of cilostazol significantly improved clinical outcomes without increasing the risk of major bleeding in patients with HPR on clopidogrel. Consequently, the relationship between clopidogrel hyporesponsiveness and thrombotic events has been probably established, especially in high-risk patients, and a well-designed large-scale RCT with optimal testing method is waarranted to evaluate the real benefit of platelet function testing.

Several questions remain unanswered. First, previous studies [12, 35] showed that HPR on aspirin was also associated with incremental thrombotic events after DES implantation. However, mounting evidence [36–38] supports that HPR on aspirin is either a very rare phenomenon or does not exist, and might due to patient noncompliance, delayed and reduced drug absorption of enteric coating, or drug-drug interactions (e.g. ibuprofen). Second, it was reported [3] that platelet function testing predicts bleeding in patients with bypass surgery. Nevertheless, other studies [12, 39] failed to show that platelet function testing reliably predicted future bleeding risk in stenting patients, in line with the present study, which might be due to the low bleeding risk of clopidogrel and coronary intervention. Therefore, the value of platelet function testing in PCI patients should be explored in further studies. Our ongoing PL-PLATELET (Ticagrelor Versus High-dose Clopidogrel in Patients With High Platelet Reactivity on Clopidogrel After PCI, NCT03078465) trial may offer further insight into the effect of platelet function testing on PCI patients.

Our study has several limitations. First, we did not assess the effect of platelet function testing guided therapy adjustment on cardiac events. Second, platelet function testing was performed at index procedure, and enrolled patients did not undergo repeated testing at later time periods. Third, Aggrestar, not VerifyNow, was used in this study due to the high cost of VerifyNow; however, Aggrestar has been shown to correlate well with VerifyNow [15]. Fourth, the study median follow-up of 4.1 years with interquartile range of 1.8 years (skewed distribution) indicates that more patients were enrolled at the early stage of this study, but the duration of follow-up was similar between two groups. Additionally, it was a real-world cohort study, and some patients changed the antiplatelet drugs themselves, although no differences were found between two groups.

## Conclusion

This prospective, propensity score-matched cohort study showed that HPR on clopidogrel was associated with incremental long-term risk of MACCE after DES implantation, mainly driven by increased all cause death and clinically driven TVR. The present study suggests potential benefits for platelet function testing, and a well-designed RCT with optimal testing method is needed to further address the real advantages of platelet function testing.

## Additional file

Additional file 1: **Figure S1.** Flowchart of study design. PCI: percutaneous coronary intervention; MAR: maximal aggregation ratio; ADP: adenosine diphosphate. (TIFF 110 kb)

## Abbreviations

ACS: Acute coronary syndrome
ADP: Adenosine diphosphate
BMI: Body mass index
CABG: Coronary artery bypass grafting
DES: Drug-eluting stent
eGFR: Estimated glomerular filtration rate
HF: Heart failure
HPR: High platelet reactivity
hsCRP: High sensitivity C reactive protein
IABP: Intra-aortic balloon pump
IVUS: Intravascular ultrasound
LTA: Light transmittance aggregation
LVEF: Left ventricular ejection fraction
MACCE: Major adverse cardiovascular and cerebrovascular events
MAR: Maximal aggregation ratio
MI: Myocardial infarction
NPR: Normal platelet reactivity
PCI: Percutaneous coronary intervention
PSM: Propensity score matching
RCT: Randomized clinical trial
TIMI: Thrombolysis in myocardial infarction
TLR: Target lesion revascularization
TVR: Target vessel revascularization
