# Supplementary figures and images for: Relationship between high platelet reactivity on clopidogrel and long-term clinical outcomes after drug-eluting stents implantation (PAINT-DES): a prospective, propensity score-matched cohort study
Source: BMC Cardiovasc Disord. 2018 May 24;18:103. doi: 10.1186/s12872-018-0841-1 (PMC5968524; doi:10.1186/s12872-018-0841-1)

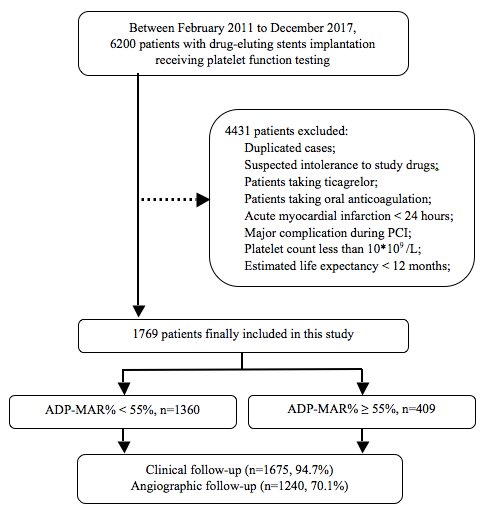

Supplement: Supplementary file 1 — Figure S1. Flowchart of study design. PCI: percutaneous coronary intervention; MAR: maximal aggregation ratio; ADP: adenosine diphosphate. (TIFF 110 kb) [file 12872_2018_841_MOESM1_ESM.tiff]
